# Can avatar affective valence determine whether virtual reality embodiment reduces implicit workplace ageism?

**DOI:** 10.3389/fpsyg.2026.1868756

**Published:** 2026-06-30

**Authors:** Ronit Elyoseph, Ehud Bodner, Doron Friedman

**Affiliations:** 1 Department of Social and Health Sciences, Bar-Ilan University, Ramat Gan, Israel; 2 Music Department, Bar-Ilan University, Ramat Gan, Israel; 3 Sammy Ofer School of Commmunications, Reichman University, Herzliya, Israel

**Keywords:** age diversity, ageism, avatar affective valence, embodiment, hiring discrimination, implicit bias, virtual reality

## Abstract

Workplace ageism disproportionately hinders older workers' employment access and organizational wellbeing. Existing interventions produce limited effects on implicit age bias, particularly among young men. VR embodiment of older adult avatars offers a promising alternative, though the role of avatar affective valence in shaping these outcomes remains poorly understood. We conducted a randomized, pre-post, between-subjects experiment (*N* = 107 young men, age range 20–30) in which participants embodied one of four VR avatars: positive (Albert Einstein), negative (Hassan Nasrallah), neutral adult, or neutral young. Implicit ageism (Age IAT) and self-esteem were assessed 1 week before and immediately after the VR session; willingness to hire a 60-year-old job candidate and explicit ageism were assessed post-intervention. Einstein's embodiment produced a significant medium-to-large reduction in implicit ageism (*d* = 0.65, *p* < 0.001); a smaller but significant reduction followed neutral adult embodiment (*d* = 0.36, *p* = 0.040). Contrary to hypotheses, negative embodiment did not increase ageism, but exploratory within-condition analyses suggested a significant decrease in self-esteem (*d* = 0.59) and a significant increase in implicit gender-career bias (*d* = 0.46). Willingness to hire was higher in the Einstein and neutral adult conditions than in the neutral young condition. Self-esteem did not moderate implicit bias change, and post-VR explicit ageism did not differ significantly across conditions, consistent with the implicit–explicit dissociation. These findings advance a meaning-based model of VR embodiment, provide controlled evidence that embodiment in a positively valenced avatar can reduce implicit workplace ageism, and document novel collateral risks of negatively valenced avatar exposure.

## Introduction

Ageism in the workplace, the stereotyping, prejudice, and discrimination directed at workers on the basis of advanced age, is one of the most prevalent and costly forms of workplace discrimination globally. In the United States, 64% of employees aged 50 and older have witnessed or experienced age discrimination at work (Perron, 2025), and applicant age has been identified among Danish employees as the single most prominent factor in employment discrimination (Qvist and Larsen, 2025). The organizational consequences are substantial. Ageism impedes productivity, diversity, and wellbeing (Kunze and De Meulenaere, 2024; Kunze et al., 2011; Marchiondo et al., 2019), while its annual economic cost in the United States alone is estimated at $63 billion (Levy et al., 2020). Ageist attitudes are documented across the organizational hierarchy, among young employees, HR professionals, and managers alike, with younger employees tending to hold particularly extreme ageist views (Bae and Choi, 2023; Harris et al., 2018). As the proportion of older workers in the labor force continues to grow, developing effective strategies to address workplace ageism has become an organizational and public health priority (World Health Organization, 2021).

The effects of existing workplace ageism prevention interventions, primarily education-based programs, awareness workshops, and intergenerational contact initiatives, are mainly demonstrated at the explicit, declarative level and tend to be short-lived (Iweins et al., 2013; Sinclair et al., 2024). This limitation is especially consequential because the mechanisms that drive age-discriminatory hiring decisions, such as preferring younger applicants or discounting older candidates' qualifications, operate largely through implicit, automatic associations that may become inaccessible to deliberate self-reflection (Kleissner and Jahn, 2020; Ratliff and Smith, 2024). Experimental evidence demonstrates that applicants listed as 60 years old consistently receive lower hiring-likelihood ratings than those listed as 20 years old, and that implicit age cues embedded in applicant profiles produce an equivalent disadvantage (Kleissner and Jahn, 2021; Krings et al., 2011). Interventions that target only conscious attitudes may leave these implicit ageist processes unchanged, resulting in a persistent gap between stated fairness and actual discriminatory behavior. In the present study, implicit workplace ageism refers to relatively automatic negative age-related associations that may shape judgments outside deliberate awareness, whereas explicit workplace ageism refers to consciously endorsed age-related beliefs and attitudes that can be reported directly. Effective workplace ageism reduction, therefore, requires approaches that can access and modify implicit associative processes, precisely where current tools fall short. In this regard, interventions enabling deeper experiential and perspective-taking change hold the greatest promise (Tassinari et al., 2022).

Virtual reality (VR) embodiment, the technology-induced illusion that a virtual body (avatar) is one's own, may suggest a distinctive pathway into this implicit level (Maister et al., 2015). By inducing a body ownership illusion through synchronization of participants' real movements with those of an avatar (visuomotor synchrony), immersive VR creates conditions for direct, first-person perspective-taking that are less achievable through imagination or instruction alone (Maister et al., 2015; Slater and Sanchez-Vives, 2016). A key mechanism underlying these effects was termed the Proteus effect, i.e., the tendency for individuals to internalize the characteristics of their avatar and adjust their self-perception, cognition, and behavior accordingly, such that the avatar's identity becomes a lens through which the self is temporarily experienced (Yee and Bailenson, 2007). Because embodiment involves experiencing the virtual body as one's own, it may influence not only explicit perspective taking but also more automatic self-other associative processes.

Studies have shown the wide social effects of embodiment in out-group avatars. Such embodiment reduces implicit racial stereotypes (Banakou et al., 2016, 2020; Groom et al., 2009; Maister et al., 2013; Peck et al., 2013). Moreover, prior work has demonstrated that embodying young participants in older adult avatars can reduce implicit age bias (Ayalon et al., 2023; Oh et al., 2016; Yee and Bailenson, 2006) and that embodiment in Einstein's avatar specifically reduces implicit ageism and improves cognitive performance among young participants (Banakou et al., 2018). These findings establish that VR embodiment can reach the implicit level at which ageist associations reside. However, they do not yet clarify whether embodiment effects are driven primarily by the avatar's age category, by its broader symbolic meaning, or by the combination of the two. Although VR shows promise for reducing ageism, most research on stereotype reduction through VR has focused on racism (see also Kishore et al., 2019). Therefore, more research is needed to reduce ageism through VR (Zou et al., 2024). Yet a critical, unanswered question remains: Does the affective valence of the embodied avatar representing the older body determine whether implicit ageism is reduced, unchanged, or increased? In the present study, affective valence refers to the overall positive or negative evaluative meaning associated with the embodied figure, including whether the avatar is perceived as positive, neutral, or negative.

This question is both theoretically and practically important. Banakou et al. (2020) demonstrated that the affective valence of the social context shapes whether VR embodiment reduces or exacerbates racial bias. However, no study has systematically manipulated the affective valence of the embodied avatar as the primary independent variable, nor has it tested these effects specifically in a workplace context. The current study is the first to do so. This question can be approached through three theoretical lenses. First, according to the Social Identity Model of Deindividuation Effects (SIDE model), reduced salience of personal identity in immersive environments can heighten attention to socially meaningful external identity cues, thereby increasing the influence of the embodied figure's symbolic characteristics on cognition and behavior (Reicher et al., 1995; Yee et al., 2009). In the present context, this framework implies that participants may respond not only to the avatar's age category, but also to the broader social meaning attached to that figure. Second, the contact hypothesis proposes that the quality and valence of intergroup contact shape prejudice reduction or maintenance, with positive contact tending to reduce bias and negative contact tending to sustain or exacerbate it (Pettigrew and Tropp, 2006; Barlow et al., 2012). Although VR embodiment is not identical to face-to-face contact, it may function as a psychologically meaningful contact-like experience in which the embodied outgroup figure becomes the medium through which social meaning is encountered. Third, body ownership accounts hold that when individuals experience a virtual body as their own, self-related associations may partially generalize to the embodied social category, thereby altering implicit attitudes toward that group (Maister et al., 2015; Slater and Sanchez-Vives, 2016). In line with the contact hypothesis, the valence of the contact enacted through embodiment should determine the direction of attitude change (Barlow et al., 2012; Pettigrew and Tropp, 2006). Moreover, the present study was not designed as a fully crossed age × valence factorial test. It was designed to compare older-adult embodiments differing in affective valence, while including a neutral young avatar as a theoretically relevant reference condition.

According to the SIDE model, the anonymity of immersive VR environments reduces the salience of participants' personal identity standards and heightens conformity to social identity cues. When these cues are provided by the embodied avatar, the avatar's characteristics become the lens through which the experience is processed (Yee et al., 2009). These perspectives converge on the prediction that embodiment effects may depend not only on whether the avatar is older, but also on whether the older figure carries positive, neutral, or negative social meaning. A positively valenced older adult avatar, such as Einstein, associated with wisdom, competence, and admirable achievements (Banakou et al., 2018), should thus activate positive associations with old age, disrupting the automatic negative associations that constitute implicit ageism (Maister et al., 2015). Such an avatar may be especially likely to weaken implicit ageism because it combines older age with socially valued identity cues and may promote both favorable contact-like meaning and positive self-generalization through embodiment. By contrast, a negatively valenced older avatar was expected to produce the opposite pattern, as it combines older age with aversive social meaning and may therefore reinforce rather than attenuate negative age-related associations. A neutral older avatar was expected to produce a weaker effect, if any, because it represents older age without the added influence of strongly positive or negative symbolic meaning. This logic also suggests that the willingness to hire an older candidate may be higher following a positively valenced older-adult embodiment than following a neutral or a negatively valenced embodiment. Accordingly, the central theoretically driven comparisons in the present study concerned the three older-adult avatars differing in valence, whereas the neutral young avatar was included primarily as a reference condition rather than as part of a fully crossed age-by-valence design.

The present study tested these predictions in a randomized mixed-design experiment with a between-subjects embodiment-condition factor and a within-subjects time factor. Young men were randomly assigned to embody one of four avatars in a workplace VR scenario modeled on a professional conference. These four conditions were selected to compare positively, negatively, and neutral older-adult embodiments against a neutral young reference condition, thereby permitting a focused test of whether the social meaning of an older embodied figure may shape age-related bias. The four avatars were Albert Einstein (positive valence adult; throughout this manuscript, “adult” refers to persons aged 60 years and older), Hassan Nasrallah (negative valence adult), a neutral adult, and a neutral young avatar.

Avatars were selected based on a validated pilot survey confirming their perceived valence. The study examined four primary hypotheses regarding young men: (H1a) embodiment in a positively valenced older-adult avatar would produce greater reductions in implicit ageism than embodiment in a neutral older-adult avatar; (H1b) embodiment in a negatively valenced older-adult avatar would produce greater increases in implicit ageism than embodiment in a neutral older-adult avatar; (H2a) embodiment in a positively valenced older-adult avatar would be associated with greater willingness to hire a 60-year-old job candidate than embodiment in the neutral conditions; and (H2b) embodiment in a negatively valenced older-adult avatar would be associated with lower willingness to hire a 60-year-old job candidate than embodiment in a neutral older-adult avatar. In addition, (H3), drawing on prior evidence that dispositional self-esteem may amplify bias reduction during outgroup embodiment (Banakou et al., 2020; Bedder et al., 2019; Galinsky and Ku, 2004), baseline self-esteem was predicted to moderate the relationship between avatar valence and implicit bias change among young men. Additionally, several outcomes were assessed exploratorily: self-esteem change, implicit gender-career bias, and explicit workplace ageism.

## Method

### Participants

The sample comprised 107 healthy young male participants, specifically recruited because young men are the demographic most strongly associated with ageist attitudes (Bodner and Lazar, 2008; Rupp et al., 2005) and least responsive to conventional ageism-reduction interventions relative to female peers (Burnes et al., 2019), making them the priority target for the present study. The target sample size was informed by an *a priori* power analysis conducted at the proposal stage. This analysis was intended for the study's primary confirmatory test, namely the 4 (Condition) × 2 (Time) mixed-design ANOVA on implicit ageism. Based on prior comparable VR studies, an initial feasibility target of 80 participants was considered realistic, whereas the formal G^*^Power analysis indicated a target of 112 participants. For a four-condition omnibus effect at α = 0.05, this target corresponds approximately to 80% power to detect an effect of *f* = 0.318 (approximately η*p*^2^ = 0.092). The final sample of 107 participants therefore approached the planned target and yielded approximately 78% power for an effect of that magnitude, indicating that the study was close to the planned power level for the primary analysis, although the effective sample sizes for some secondary and exploratory analyses were smaller. Participants were randomly assigned to one of four experimental conditions: Einstein (*n* = 29), Nasrallah (*n* = 26), Neutral Adult (*n* = 26), and Neutral Young (*n* = 26). Inclusion criteria were: male, age 20–30, Hebrew-speaking, and prior work experience. Participants with a history of epilepsy were excluded following Banakou et al. (2018, 2020). The final sample ranged in age from 20 to 30 years (*M* = 24.89, *SD* = 2.04). Educationally, 90.7% were current undergraduate students and 9.3% were BA graduates. Regarding religiosity, 82.2% identified as secular and 17.8% as traditional or moderately religious. Most participants (58.9%) were currently employed, primarily in salaried positions. However, all participants had prior years of work experience (*M* = 4.71, *SD* = 3.15), and 85% reported some managerial experience (*M* = 2.28, *SD* = 0.82, on a 1–4 scale: 1 = *not at all*, 2 = *a little*, 3 = *quite a lot*, 4 = *a lot*), making the sample appropriate for the willingness-to-hire task (cf. Krings et al., 2011; Fritzsche and Marcus, 2013). The study was approved by the Reichman University IDC Institutional Review Board (no. 17-02-22), and all participants provided written informed consent and received 30 NIS compensation. Sample characteristics (continuous) and baseline equivalence variables across experimental conditions are presented in Table 1. No significant between-condition differences were found on any demographic or pre-intervention variable, supporting successful randomization.

**Table 1:** Sample characteristics and baseline equivalence across experimental conditions, presented as mean (SD).

Variable	Einstein (*n* = 29)	Nasrallah (*n* = 26)	Neutral adult (*n* = 26)	Neutral young (*n* = 26)	Total (*N* = 107)	*Statistic*	*p*
Age	25.07 (2.70)	24.81 (1.88)	24.50 (1.56)	25.15 (1.83)	24.89 (2.04)	*WelchF*_(3, 57)_ = 0.72	0.543
Years of work experience	4.79 (3.81)	4.54 (2.40)	5.19 (3.42)	4.31 (2.83)	4.71 (3.15)	*F*_(3, 103)_ = 0.37	0.775
Managerial experience	2.24 (0.83)	2.38 (0.90)	2.23 (0.65)	2.27 (0.92)	2.28 (0.82)	*F*_(3, 103)_ = 0.19	0.903
Prior VR experience	2.48 (0.99)	2.65 (1.13)	2.31 (1.16)	2.58 (0.99)	2.50 (1.06)	*F*_(3, 103)_ = 0.51	0.676
Implicit ageism (T1)	0.78 (0.37)	0.78 (0.31)	0.76 (0.37)	0.73 (0.35)	0.76 (0.35)	*F*_(3, 103)_ = 0.13	0.943
Self-esteem (T1)	3.23 (0.41)	3.25 (0.61)	3.19 (0.44)	3.24 (0.54)	3.23 (0.50)	*F*_(3, 103)_ = 0.07	0.974
Implicit gender-career bias (T1)^*^	0.43 (0.26)	0.27 (0.40)	0.32 (0.38)	0.45 (0.24)	0.37 (0.33)	*F*_(3, 59)_ = 1.11	0.354

T1 = first session, before the VR experience. Values are means (*SD*). Welch's *F* was reported for age because Levene's test indicated heterogeneity of variances (*p* = 0.034).

^*^For Implicit Gender-Career Bias (T1), the overall total is based on a reduced subsample (*n* = 63; Einstein *n* = 18, Nasrallah *n* = 16, neutral adult *n* = 15, neutral young *n* = 14), because this measure was added after data collection had begun.

### Research design

The study employed a 4 (Embodiment Condition: Einstein, Nasrallah, Neutral Adult, Neutral Young) × 2 (Time: Before, After VR experience) mixed-design experiment, with embodiment condition as the between-subjects factor and time as the within-subjects factor. This was a theory-driven four-condition comparative design rather than a fully crossed age × valence factorial design. More specifically, the design was intended to compare older-adult embodiments that differed in affective valence (positive, negative, neutral), with a neutral young avatar as a reference condition. Participants were randomly assigned to conditions using a random-number allocation procedure. Two sessions, separated by 1 week, were conducted to minimize IAT practice effects.

### VR experience and avatar construction

**Figure 1:**
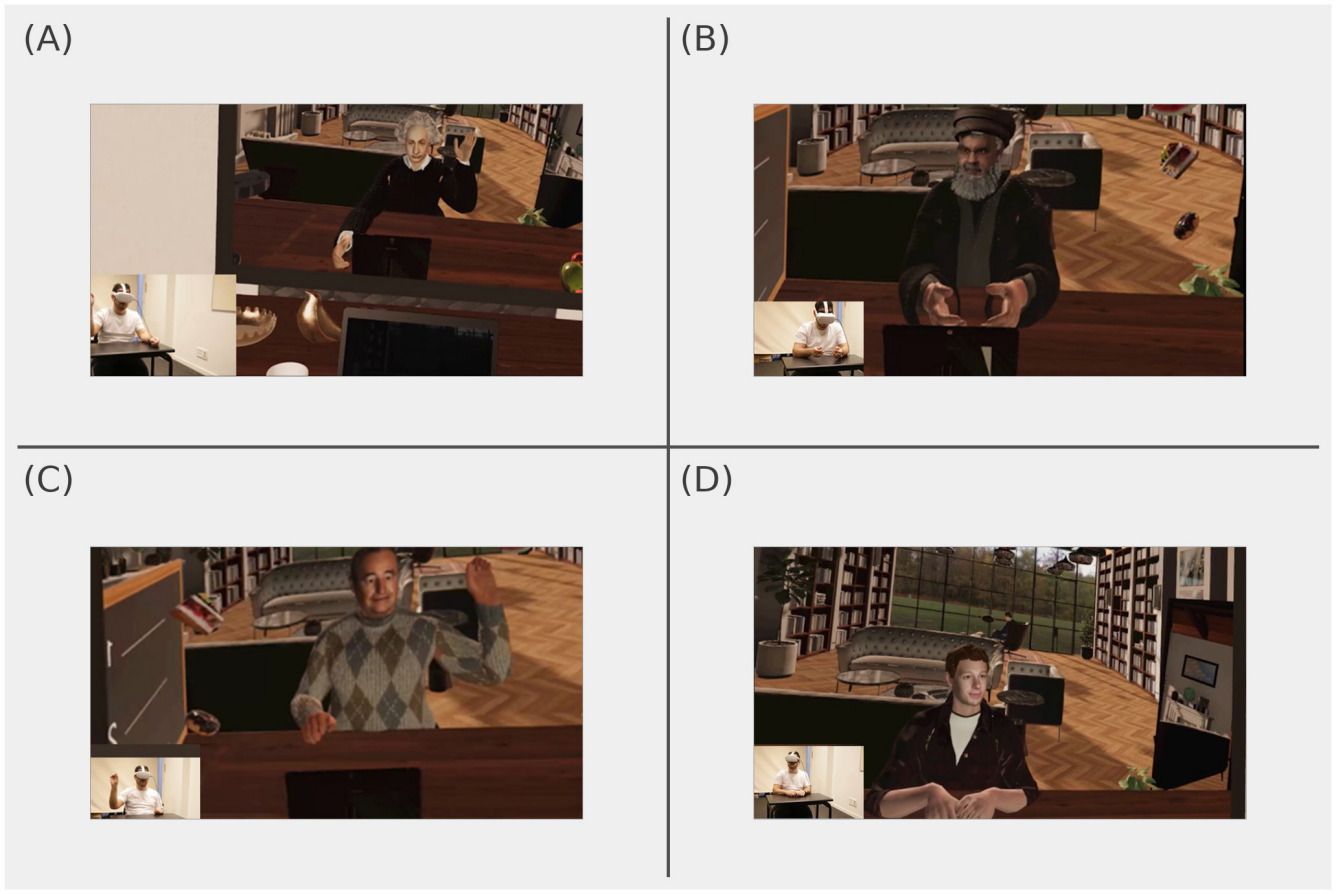
An illustration of the embodiment in the four conditions. **(A)** Einstein. **(B)** Nasrallah. **(C)** Neutral adult. **(D)** Neutral young.

Participants wore an Oculus Quest 2 headset and sat at a desk, mirroring their real posture. The virtual environment depicted a professional conference room, a neutral, non-threatening, workplace-relevant scenario. The scenario was designed to be neutral and non-threatening, so that any effects on implicit ageism would be attributable to avatar valence rather than environmental valence. This is a methodological precaution established by Peck et al. (2013) and confirmed by Banakou et al. (2020), who demonstrated that negative-valence contexts reverse the bias-reduction effect of outgroup embodiment. The virtual environment was developed using the Unity 3D engine, and 3D avatars were modeled from 2D images using Character Creator (Reallusion, n.d.; Figure 1). Body ownership was induced through visuomotor synchrony: participants saw their avatar move in synchrony with their movements via two virtual mirrors, inducing the illusion that the virtual body was their own (Slater and Sanchez-Vives, 2016).

Avatar selection followed a two-step procedure. First, a brainstorming session with 20 young men identified well-known adult figures (aged 60 and older) perceived as positive or negative. Second, an online validation survey of 37 young men aged 20–35 (*M* = 28.3, *SD* = 4.3) confirmed the affective valence of eight candidates: Four positive (Albert Einstein, Bill Clinton, George Clooney, and the Dalai Lama) and four negative (Hassan Nasrallah, Joseph Stalin, Harvey Weinstein, and Jeffrey Epstein). Each one was rated on a 1–7 scale from a photograph. Albert Einstein received the highest positive valence rating (*M* = 6.47, *SD* = 0.70), and Hassan Nasrallah received the highest negative valence rating (*M* = 1.46, *SD* = 1.1) on a 1–7 scale. Neutral adult and neutral young avatars were adapted from Yee and Bailenson (2006).

### Procedure

The experiment comprised two laboratory sessions. During Session 1 (T1), held 1 week before the VR experience, participants completed socio-demographic questionnaires, self-report measures, and pre-VR Implicit Association Tests (IATs) assessing ageism and gender-career bias. The assessments lasted around 40 min per participant. During Session 2 (T2), participants underwent the VR embodiment experience, which included an initial embodiment phase. Participants were instructed to perform a series of simple movements while viewing their avatar from a first-person perspective in two virtual mirrors and to complete easy tasks (selecting food items from a virtual menu) within the environment to strengthen the body ownership illusion (about 4 min). Participants' head and hand movements were tracked in real time and synchronously mapped onto the avatar, creating visuomotor synchrony throughout the embodiment phase. This stage was followed immediately by post-VR IATs, the willingness-to-hire task, the embodiment and presence scale, and additional questionnaires, which lasted around 60 min per participant. The 1-week interval between sessions was intended to minimize practice effects on the IAT.

### Measures

#### Implicit age bias (age IAT)

Implicit age bias was the primary outcome, assessed using the Implicit Association Test (IAT) (Greenwald et al., 1998), which measures the strength of automatic associations between social categories (young vs. older adults) and evaluative attributes (pleasant vs. unpleasant) by comparing response times across congruent and incongruent categorization trials (Nosek et al., 2005, 2007). Faster categorization of older adults with negative adjectives and young adults with positive adjectives indicates stronger implicit age bias. The IAT D-score was computed following standard procedures (Nosek et al., 2005). Although criticisms of the IAT have been raised (Schimmack, 2021), there is less consensus on these objections, and the measure remains widely used in social psychology research. There is robust support for the IAT's reliability and validity in age-bias contexts (Jelenec and Steffens, 2002; Ratliff and Smith, 2024). Moreover, the IAT has been employed as the standard implicit measure in prior VR embodiment studies that the current design builds on (Banakou et al., 2018, 2020; Peck et al., 2013). This test was administered at T1 and T2, and the T2–T1 difference score served as the primary dependent variable for Hypothesis 1. Implicit ageism was treated as the primary dependent variable because the study was designed to examine whether VR embodiment could modify automatic age-related associations, which were considered the most theoretically relevant mechanism for workplace bias in the present context.

#### Willingness to hire an adult employee

Willingness to hire an adult employee was the primary behavioral outcome. Participants were asked to assume the role of a Human Resources (HR) manager and review the resume of a 60-year-old male job candidate applying for a customer-service supervisory position (adapted from Krings et al., 2011; Fritzsche and Marcus, 2013). They rated their willingness to hire on four items (e.g., “This candidate should get the job”) using a 7-point Likert scale. Mean scores were computed (Cronbach's α = 0.80). This measure was administered at T2 (see Data Sheet 1).

#### Embodiment and presence scale

As a manipulation check for VR effectiveness, participants completed an 8-item scale measuring body ownership, agency, and presence (Banakou et al., 2020), with presence items adapted for the current scenario following Neyret et al. (2020) and a validated Hebrew translation based on Landau et al. (2020), rated on a 7-point scale (1 = strongly disagree, 7 = strongly agree). In the present study, this combined measure was intended to assess whether participants experienced the virtual body and environment as sufficiently immersive for the embodiment manipulation to be meaningful. Because the study did not formulate separate hypotheses regarding body ownership, agency, or presence, these related experiential indicators were used as a single overall manipulation-check index rather than analyzed as distinct outcome variables. Two items showed low item-total correlations and were removed: (“I felt as if I had two bodies” and “I felt that my virtual body resembled my body in terms of shape, skin tone, or other visual characteristics”). The final 6-item scale demonstrated good reliability (Cronbach's α = 0.76), supporting its use as a single manipulation-check index of overall immersive embodiment quality (see Data Sheet 1).

As additional manipulation checks, participants rated perceived embodied avatar comfort on a scale from 1 (very uncomfortable) to 7 (very comfortable), and perceived avatar affective valence on a scale from 1 (very negative) to 10 (very positive). These measures were used to verify that the avatars were experienced in line with their intended valence.

#### Dominant avatar characteristic

To provide qualitative evidence for a manipulation check following the SIDE model predictions regarding avatar identity cue salience, participants responded to a single open-ended item: “What is the most salient characteristic of the figure in which you saw yourself during the VR experience?” Responses were provided in free text and subsequently coded into six predefined thematic categories: Wisdom (e.g., wise, genius, intelligent), Aggression (e.g., evil, violent, aggressive), General Positivity (broadly positive descriptors), General Negativity (broadly negative descriptors), Old Age (explicit age references), and Young Age (youth-related descriptors). Each category was coded dichotomously per response (0 = not mentioned; 1 = mentioned), and categories were non-mutually exclusive. Prevalence rates were calculated as the proportion of participants endorsing each category within each condition. Coding was conducted by one trained researcher using predefined, behaviorally anchored categories to minimize interpretive ambiguity. This item was administered to the near-full sample (*N* = 101).

#### Self-esteem

The Rosenberg Self-Esteem Scale (Rosenberg, 1965) was administered at T1 as a pre-specified moderator of VR embodiment effects on implicit ageism (H3; Galinsky and Ku, 2004; Bedder et al., 2019; Banakou et al., 2020). A second T2 administration was added after approximately one-third of participants (*N* = 67) had completed the protocol to enable an exploratory repeated assessment of self-esteem scores following the VR experience. This addition was motivated by the possibility that immersive embodiment in a socially salient avatar might be associated with short-term fluctuations in self-evaluative responses, even when assessed with a global self-esteem instrument. Because the Rosenberg scale is typically used to measure global self-esteem, this repeated administration was interpreted with caution and not as a validated measure of state self-esteem. The 10-item, 4-point scale demonstrated high reliability at T1 (Cronbach's α = 0.85) and acceptable reliability at T2 (Cronbach's α = 0.78).

#### Exploratory measures

Two exploratory outcomes were added after approximately one-third of participants had completed the protocol. These measures were treated as exploratory because the original confirmatory design focused on implicit age bias as the primary target of the embodiment manipulation, whereas explicit workplace ageism and broader non-age outcomes were included only to examine possible additional or collateral patterns. Accordingly, the corresponding analyses should be interpreted as hypothesis-generating rather than confirmatory and are based on reduced sample sizes (*N* = 60–67). The experimental manipulation, recruitment procedures, randomization, and primary outcome assessment remained identical across the entire data-collection period. The exploratory measures were: (1) Explicit Ageism (SIC Scale for the Workplace): A workplace-adapted version of the Succession, Identity, and Consumption scale (North and Fiske, 2013) was developed and validated in a pre-experiment survey (*N* = 169; Cronbach's α = 0.89). Post-VR administration used the adapted 20-item version (Cronbach's α = 0.81). (2) Implicit Gender-Career Bias (Gender-Career IAT): An IAT measuring the association between gender and career/family roles, administered at T1 and T2, assessed whether VR embodiment influenced gender stereotyping beyond the ageism domain.

### Data analysis

All analyses were conducted in IBM SPSS Statistics v31. Responses to the dominant avatar characteristic item (*N* = 101) were coded into predefined thematic categories and analyzed descriptively, with frequencies and percentages reported for each condition. Distributional assumptions were screened by examining skewness and kurtosis for the primary study variables. Skewness and kurtosis values were below 1 in absolute value, indicating no substantial deviation from normality. To examine H1a and H1b, a 4 × 2 mixed-design ANOVA with repeated measures on Time was conducted, followed by focused comparisons and within-condition paired-samples t-tests to clarify the pattern of change across the four embodiment conditions. To examine H2a and H2b, a one-way ANOVA was used to test between-condition differences in willingness to hire, followed by theory-guided comparisons corresponding to the differentiated predictions. For Hypothesis 3, a moderation analysis was conducted using Hayes' PROCESS macro v5.0 (Hayes, 2022), Model 1, with self-esteem mean-centered and the neutral adult condition as the reference category. Statistical significance was evaluated at α = 0.05. Given the directional nature of the hypotheses, within-condition paired *t*-tests (H1) and pairwise comparisons (H2) were one-tailed; two-tailed *p*-values are also reported for transparency. For between-group pairwise comparisons (H2), Cohen's *d* was computed as the mean difference between conditions divided by their pooled standard deviation. Baseline self-esteem (T1) was mean-centered and entered as the moderator in H3. When multiple pairwise comparisons were performed, Bonferroni corrections were applied. Effect sizes are reported throughout: partial η^2^ for ANOVAs and Cohen's *d* for *t*-tests. Following conventional guidelines (Lakens, 2013), *d* = 0.20, η^2^ = 0.01 indicates small; *d* = 0.50, η^2^ = 0.06 medium; and *d* = 0.80, η^2^ = 0.14 large effects. Missing data were handled using the default listwise procedure within each analysis. Therefore, the valid sample size varied across analyses depending on data availability. This issue was particularly relevant to several exploratory measures introduced after data collection began, as described in the Method Section.

## Results

### Preliminary analyses: randomization and manipulation checks

Of the 107 participants, all contributed Age-IAT data. Six had missing post-VR data on some measures (resulting in *N* = 101 for willingness-to-hire and manipulation-check analyses). Exploratory measures were added mid-study and are available only for subsamples (*N* = 60–67), as described in the Method section. Randomization adequacy was confirmed: no significant pre-VR (T1) differences were found across the four conditions in implicit age bias, self-esteem, gender-career bias, or any demographic variable (all *ps* > 0.354; see Table 1). Manipulation checks confirmed that avatars were perceived as intended (Table 2). Perceived affective valence differed significantly across conditions, *F*_(3, 97)_ = 113.45, *p* < 0.001, η^2^ = 0.778, with Einstein rated most positive (*M* = 9.11, *SD* = 1.17) and Nasrallah most negative (*M* = 1.48, *SD* = 0.82). Perceived avatar age also supported the manipulation: both were perceived as older adults, but Einstein was perceived as older than Nasrallah, *t*_(51)_ = 4.43, *p* < 0.001 (Einstein: *M* = 71.89, *SD* = 6.42; Nasrallah: *M* = 64.40, *SD* = 5.84). At the same time, the fact that the Einstein and Nasrallah conditions differed not only in affective valence but also in perceived avatar age complicates the interpretation of their contrast as a purely valence comparison. Perceived avatar comfort was significantly lower in the Nasrallah condition than in all others (all *ps* < 0.021). Notably, the Embodiment and Presence Scale did not differ across conditions, *F*_(3, 97)_ = 0.58, *p* = 0.627, confirming that immersion quality was equated and that between-condition differences in outcomes were not attributable to differential VR effectiveness. The mean of this variable, measured on a 1–7 scale, indicated that, across all conditions, participants experienced agency, presence, and ownership of a virtual body (embodiment) at a level in the upper half of the scale.

**Table 2:** Manipulation checks and operational validity by experimental condition.

Variable	Einstein	Nasrallah	Neutral adult	Neutral young	Statistic	*p*
Embodiment and presence scale	4.38 (1.20)	4.60 (1.01)	4.24 (0.99)	4.51 (0.89)	*F*_(3, 97)_ = 0.58	0.627
Perceived avatar comfort	5.11 (1.37)	3.04 (0.94)^*^	4.15 (1.69)	4.64 (1.14)	*F*_(3, 97)_ = 11.54	< 0.001
Perceived avatar affective valence	9.11 (1.17)	1.48 (0.82)^*^	6.96 (2.18)	6.23 (1.66)	*F*_(3, 97)_ = 113.45	< 0.001

Values are means (*SD*). Perceived avatar comfort was rated from 1 (*very uncomfortable*) to 7 (*very comfortable*); affective valence was rated from 1 (*very negative*) to 10 (*very positive*). ^*^Nasrallah differed significantly from all other conditions (Bonferroni-adjusted *p* ≤ 0.020).

### Dominant avatar characteristics

Open-ended responses to the dominant-avatar-characteristic item supported the intended identity-cue pattern. More specifically, Einstein was primarily associated with wisdom and positivity, Nasrallah with aggression and negativity, and the neutral conditions were differentiated mainly by age-related descriptors. Full category frequencies are reported in the Supplementary Table S1.

**Figure 2:**
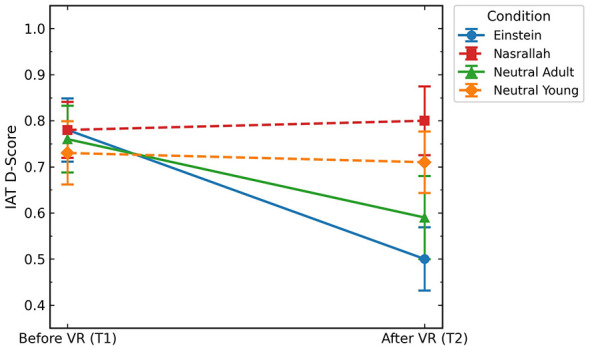
Changes in implicit ageism (age IAT D-score) by experimental condition and time. Error bars represent ±1 standard error of the mean (SEM).

**Hypothesis 1: Changes in Implicit Ageism by Condition and Time (H1a and H1b)**. A 4 (Condition) × 2 (Time) mixed-design ANOVA revealed a significant Condition × Time interaction, *F*_(3, 103)_ = 3.45, *p* = 0.019, η^2^ = 0.091, indicating that changes in implicit ageism over time differed across embodiment conditions. Within-condition paired *t*-tests clarified the pattern (see Table 3 and Figure 2). Embodiment in the Einstein avatar produced a significant, medium-to-large reduction in implicit ageism, *t*_(28)_ = 3.51, *p* < 0.001, *d* = 0.65, η^2^ = 0.306. A smaller reduction was also observed in the Neutral Adult condition *t*_(25)_ = 1.83, *p* = 0.040, *d* = 0.36, η^2^ = 0.118. In contrast, no significant change was found in the Nasrallah condition, *t*_(25)_ = 0.20, *p* = 0.421, *d* = −0.04, η^2^ = 0.002, or in the Neutral Young condition, *t*_(25)_ = −0.46, *p* = 0.325, *d* = 0.09, η^2^ = 0.008. H1a received partial support in that embodiment in the positively valenced older-adult avatar was associated with a pronounced reduction in implicit ageism, whereas the neutral older-adult condition showed only a smaller reduction.

**Table 3:** Pre- to post-VR changes in primary and exploratory repeated-measures outcomes by experimental condition.

Outcome and condition	*n*	Before *M* (*SD*)	After *M* (*SD*)	Change	*t* (df)	*p*	*d*
Implicit ageism (age IAT)							
Einstein	29	0.78 (0.37)	0.50 (0.37)	−0.28	−3.51 (28)	< 0.001	0.65
Neutral adult	26	0.76 (0.37)	0.59 (0.46)	−0.17	−1.83 (25)	0.040	0.36
Nasrallah	26	0.78 (0.31)	0.80 (0.38)	0.02	0.20 (25)	0.421	0.04
Neutral young	26	0.73 (0.35)	0.71 (0.34)	−0.02	−0.46 (25)	0.325	0.09
Self-esteem							
Einstein	19	3.31 (0.42)	3.27 (0.43)	−0.04	0.31 (18)	0.763	0.07
Neutral adult	16	3.24 (0.34)	3.26 (0.36)	0.01	−0.20 (15)	0.844	0.05
Nasrallah	16	3.27 (0.57)	2.89 (0.58)	−0.38	2.37 (15)	0.032	0.59
Neutral young	16	3.31 (0.44)	3.21 (0.54)	−0.10	0.94 (15)	0.362	0.24
Implicit gender-career bias							
Einstein	18	0.43 (0.26)	0.31 (0.33)	−0.12	1.32 (17)	0.101	0.31
Neutral adult	15	0.32 (0.38)	0.41 (0.40)	0.09	−0.83 (14)	0.210	0.22
Nasrallah	16	0.27 (0.40)	0.48 (0.30)	0.21	−1.83 (15)	0.043	0.46
Neutral young	14	0.45 (0.24)	0.35 (0.44)	−0.10	0.77 (13)	0.227	0.21

*t*-tests are within-condition paired-samples comparisons. For implicit ageism, negative change values indicate a reduction in implicit ageism. For self-esteem, negative change values indicate a decrease in self-esteem. For implicit gender-career bias, positive change values indicate an increase in implicit gender-career bias. Reduced *n* values for self-esteem and implicit gender-career bias reflect exploratory measures added after data collection had begun.

**Hypothesis 2: Willingness to Hire an Adult Employee (H2a and H2b)**. A one-way ANOVA revealed no statistically significant overall condition effect on willingness to hire, *F*_(3, 97)_ = 1.98, *p* = 0.121, η^2^ = 0.058, although the effect size was in the medium range. Pairwise comparisons, using one-tailed tests given the directional predictions, yielded two nominally significant results. Willingness to hire was descriptively higher in the Einstein condition (*M* = 5.99, *SD* = 0.87) than in the Neutral Young condition (*M* = 5.52, *SD* = 0.79), *t*_(48)_ = 1.97, *p* = 0.027, *d* = 0.56, η^2^ = 0.075, and in the Neutral Adult condition (*M* = 5.96, *SD* = 0.71) compared to the Neutral Young condition, *t*_(46)_ = 2.03, *p* = 0.024, *d* = 0.59, η^2^ = 0.082. The Nasrallah condition fell between these values (*M* = 5.75, *SD* = 0.63) and did not differ significantly from the Neutral Adult condition, suggesting that negative valence did not translate into greater reluctance to hire an older candidate. At the omnibus level, Hypothesis 2 was not supported. H2a received only limited descriptive support: willingness-to-hire scores were higher in the positively valenced older-adult condition than in the neutral young condition, but the overall condition effect was not significant. H2b was not supported because the negatively valenced older-adult condition did not show a lower willingness to hire than the neutral older-adult condition.

**Hypothesis 3: Self-Esteem as a Moderator**. A moderation analysis using PROCESS Model 1 (Hayes, 2022) did not support the prediction that baseline self-esteem (T1) would moderate the relationship between avatar valence and change in implicit ageism. The overall model was not significant, Δ*R*^2^ = 0.118, *F*_(7, 99)_ = 1.90, *p* = 0.078. The omnibus test of the condition × self-esteem interaction was non-significant, Δ*R*^2^ = 0.010, *F*_(3, 99)_ = 0.35, *p* = 0.787, and no individual interaction term approached significance (all *ps* > 0.326). Hypothesis 3 was not supported.

### Exploratory findings

#### Exploratory change in self-esteem scores by condition

**Figure 3:**
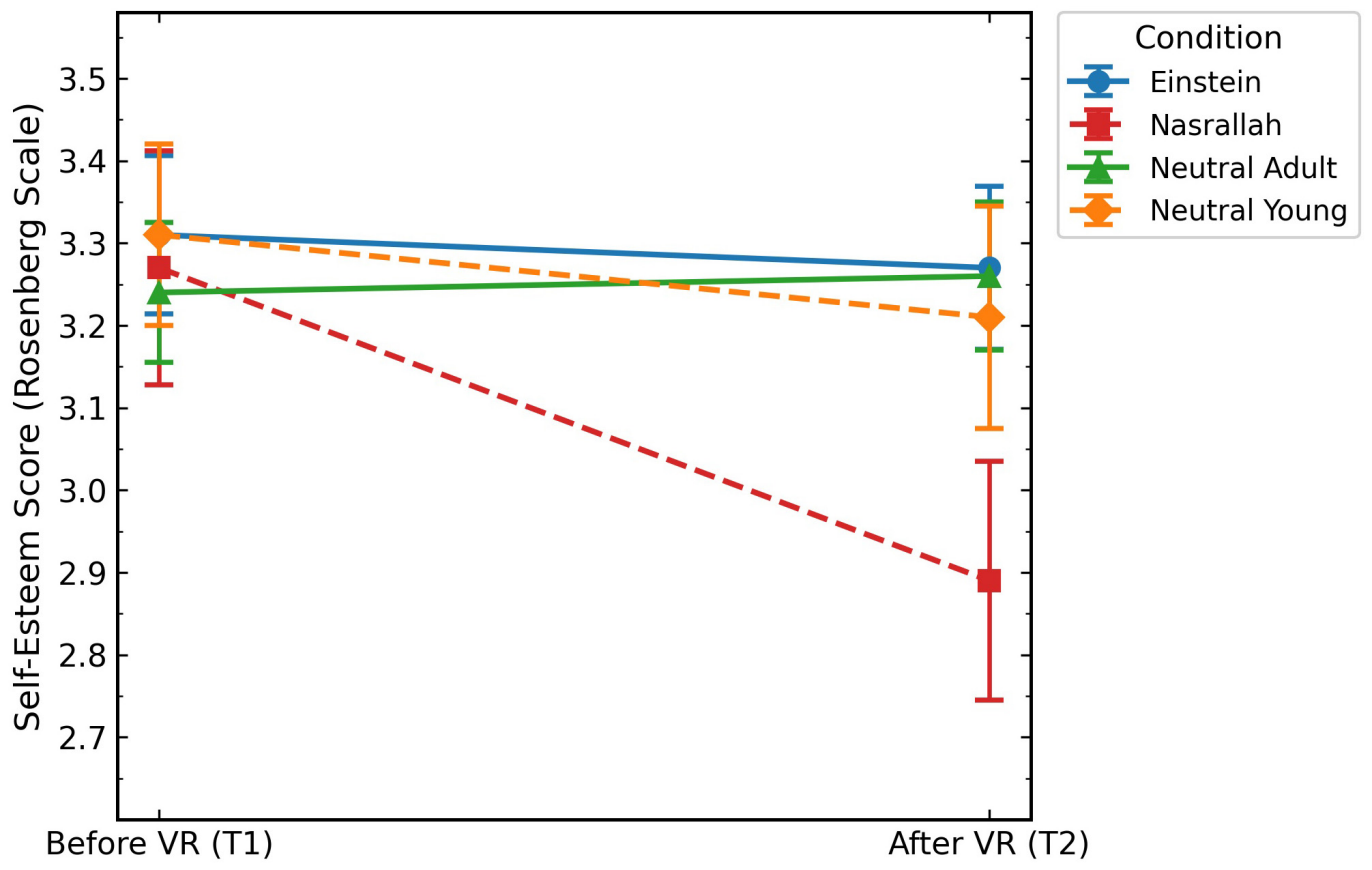
Changes in self-esteem by experimental condition and time. Error bars represent ±1 standard error of the mean (SEM).

A 4 × 2 mixed-design ANOVA on self-esteem revealed a significant main effect of Time, *F*_(1, 63)_ = 4.47, *p* = 0.039, η^2^ = 0.066, indicating a general decrease in self-esteem from T1 to T2. The Condition × Time interaction was not significant, *F*_(3, 63)_ = 2.09, *p* = 0.110, η^2^ = 0.091, though the effect size was medium. Exploratory within-condition analyses suggested a decrease in self-esteem in the Nasrallah condition from *M* = 3.27 (*SD* = 0.57) at T1 to *M* = 2.89 (*SD* = 0.58) at T2, *t*_(15)_ = 2.37, *p* = 0.032, *d* = 0.59, η^2^ = 0.272. No significant changes were observed in the Einstein condition, from *M* = 3.31 (*SD* = 0.42) to *M* = 3.27 (*SD* = 0.43), *d* = 0.07; the Neutral Adult condition, from *M* = 3.24 (*SD* = 0.34) to *M* = 3.26 (*SD* = 0.36), *d* = 0.05; or the Neutral Young condition, from *M* = 3.31 (*SD* = 0.44) to *M* = 3.21 (*SD* = 0.54), *d* = 0.24. Changes in self-esteem across conditions are presented in Figure 3.

#### Implicit gender-career bias change by condition

A 4 × 2 mixed-design ANOVA on gender-career IAT scores yielded a non-significant Condition × Time interaction, *F*_(3, 59)_ = 2.15, *p* = 0.104, η^2^ = 0.098, indicating a medium-to-large effect size. Exploratory within-condition analyses suggested a possible increase in implicit gender-career bias in the Nasrallah condition, from *M* = 0.27 (*SD* = 0.40) at T1 to *M* = 0.48 (*SD* = 0.30) at T2, *t*_(15)_ = 1.83, *p* = 0.043, *d* = 0.46, η^2^ = 0.183. No significant changes were observed in the Einstein condition, from *M* = 0.43 (*SD* = 0.26) to *M* = 0.31 (*SD* = 0.33), *p* = 0.101, *d* = 0.31; *p* = 0.763, *d* = 0.07, η^2^ = 0.005, the Neutral Adult condition, from *M* = 0.32 (*SD* = 0.38) to *M* = 0.41 (*SD* = 0.40), *p* = 0.210, *d* = 0.22, *p* = 0.844, η^2^ = 0.003; or the Neutral Young condition, from *M* = 0.45 (*SD* = 0.24) to *M* = 0.35 (*SD* = 0.44), *p* = 0.227, *d* = 0.21.

Finally, to examine differences in explicit ageism at work (SIC) across the four experimental conditions (Einstein, Nasrallah, Neutral Adult, Neutral Young), a one-way analysis of variance (ANOVA) was conducted. The analysis revealed no significant effect of the experimental condition on explicit ageism, *F*_(3, 56)_ = 0.31, *p* = 0.820, with a very small effect size (η^2^ = 0.016). Bonferroni-corrected *post hoc* comparisons indicated that none of the pairwise comparisons between conditions were significant (all *ps* = 1.000). Thus, explicit ageism scores did not differ between participants embodied in the positive avatar (Einstein), the negative avatar (Nasrallah), or the two neutral avatars (Neutral Adult and Neutral Young).

## Discussion

This study examined whether the affective valence of an embodied older-adult avatar, rather than its apparent age alone, influences changes in implicit workplace ageism among young men. The findings carry implications for research on intergroup bias, VR embodiment, and the reduction of workplace ageism, though they warrant cautious interpretation. To our knowledge, this is the first study to manipulate avatar affective valence as the primary independent variable in a VR ageism experiment and to test these effects in a workplace-relevant context that also included a hiring-intention outcome. The results suggest that avatar valence may matter for embodiment-related attitude change, but its effects are neither uniform nor straightforward across outcomes. The open-ended avatar-characteristic responses fit the idea that salient identity cues of the embodied figure shaped participants' experience. More tentatively, the findings point to a meaning-based account of embodiment effects on implicit bias. Finally, the pattern observed in the Nasrallah condition raises the possibility of collateral psychological costs—particularly regarding self-esteem and gender-career bias—that may have ethical implications for VR intervention design. We discuss these issues in turn below.

### The potency of positive valence

The study's central finding was a significant, medium-to-large reduction in implicit ageism among participants embodied as Albert Einstein. This replicates and extends Banakou et al. (2018), who first demonstrated that embodying Einstein reduces implicit age bias. It additionally extends Banakou et al. (2020), who established that the affective valence of the social context determines the direction of the embodiment effect on implicit bias. The present study confirms this principle and makes a few novel contributions to this finding.

First, it demonstrates this effect in a workplace context, using a professional conference scenario and linking it to hiring intentions, the domain where age discrimination is most consequential. Additionally, it establishes avatar valence as the primary mechanism by contrasting Einstein with three other avatar conditions and showing that valence, not age alone, drives the effect. Finally, it offers a potentially theoretically grounded account of the effect through the SIDE model and the body ownership illusion (Reicher et al., 1995; Yee et al., 2009; Maister et al., 2015; Slater and Sanchez-Vives, 2016).

The theoretical mechanism most consistent with this finding integrates two accounts. According to the body ownership illusion framework (Maister et al., 2015), when visuomotor synchrony induces a sense that the virtual body is one's own, the self's positive implicit associations generalize to the outgroup represented by the avatar. This generalization holds because the self typically carries strong positive valence, and most people hold positive implicit associations with themselves (Greenwald and Banaji, 1995). When the embodied figure is an older adult, these self-positivity associations extend to the older-adult category, weakening the automatic negative associations that constitute implicit ageism. The SIDE model (Reicher et al., 1995; Postmes and Spears, 1998) explains why avatar valence specifically matters. The anonymity and deindividuation of the VR environment reduce the salience of participants' personal identity standards and increase attention to external situational cues, such as the avatar's identity characteristics (Yee et al., 2009). When the avatar is Einstein, these cues are unambiguously positive and associated with wisdom, genius, and scientific achievement. These cues become the dominant lens through which “old age” is encountered, disrupting the automatic young equals positive or old equals negative associations measured by the age IAT and consistent with the positive contact hypothesis (Pettigrew and Tropp, 2006; Marques et al., 2020).

Our findings are also consistent with Marques et al. (2020), who found that the quality of contact with adults and the negative or positive valence of this contact is the strongest influence on ageism, thereby suggesting that in VR embodiment, the perceived meaning, attributes, and valence of the embodied virtual figure can influence the change in ageism even in a work environment. This account is further supported by Hasler et al. (2017), who found that the extent to which white participants positively evaluated the black virtual character they embodied was associated with a greater reduction in implicit racial bias, a pattern parallel to the Einstein effect observed here. This interpretation should nevertheless be qualified by the fact that Einstein was also perceived as older than Nasrallah. Accordingly, the stronger effect observed in the Einstein condition cannot be unambiguously attributed to positive valence alone, as it may also partly reflect a more salient older-age cue.

Direct behavioral evidence for the SIDE model's predictions was provided by the open-ended data on dominant avatar characteristics (see Supplementary Table S1). Participants in the Einstein condition spontaneously generated wisdom-related descriptors (50.0%) far more than those in any other condition (0–3.8%), while Nasrallah participants generated aggression-related descriptors (24.0%) exclusively. This near-total condition specificity in identity-cue salience confirms that deindividuation in VR causes participants to attend to and internalize the avatar's defining characteristics, consistent with both the SIDE model and recent work by Martin Coesel et al. (2025), which demonstrates that avatar identification mediates the Proteus effect.

### The asymmetric valence effect

Contrary to H1b, embodiment in the Nasrallah avatar did not increase implicit ageism relative to the neutral older-adult condition. This asymmetry, in which positive valence reduces ageism but negative valence does not increase it, is theoretically informative. We propose that the mechanism is moral avatar rejection. The manipulation checks confirmed that Nasrallah was experienced with significantly lower comfort than in all other conditions. Rather than conforming to the avatar's identity cues as the SIDE model predicts, participants appear to have actively disidentified with the avatar, psychologically distancing themselves from an identity that constituted a severe affront to their moral self-image. Lower avatar comfort may have contributed to this reduced self-relevance and thus to the null ageism effect.

This interpretation fits research showing that avatar trait desirability functions as a boundary condition of the Proteus effect. When avatars are associated with socially undesirable characteristics, participants disidentify rather than conform (McCain et al., 2018; Praetorius and Görlich, 2020), and the Proteus effect is stronger the closer participants feel to their avatar (Ratan et al., 2020).

A second possible explanation is that participants may already have entered the study with relatively high levels of implicit ageism, leaving little room for further increase. In other words, the null effect in the Nasrallah condition may reflect a ceiling-like pattern rather than the absence of negative meaning. This interpretation is consistent with prior research showing that young adults tend to hold particularly strong implicit age bias and often begin with relatively elevated baseline levels of ageism (Chopik and Giasson, 2017; North and Fiske, 2012).

The finding also has cultural and temporal specificity. The study was conducted during the Israel–Hezbollah war (late 2023–2024), a period during which Nasrallah's association with a direct existential threat to Israeli participants was at its peak and before his death in September 2024. This timing likely amplified the moral aversion beyond what a less politically charged negative figure would produce, and participants may have perceived Nasrallah primarily as an enemy rather than as an old man. The null finding for ageism-increase may therefore be specific to extreme moral repudiation, and a moderately negative older avatar might yield different results in other contexts or cultures.

These findings are more consistent with a meaning-based than a purely perceptual interpretation of VR embodiment effects. Participants did not seem to encounter “old age” abstractly, but rather as fused with meaning, moral valence, and social significance. The Einstein condition fused old age with wisdom and positive distinctiveness; the neutral condition allowed engagement with age without defensive resistance; and the Nasrallah condition activated aversion so strongly that constructive identification was precluded. The asymmetry—positive avatar embodiment producing significant reductions in ageism while negative avatar exposure does not increase it—has important implications for intervention design. While the upside of positive avatar embodiment is evident, the downside risk of negative avatar exposure may be more limited than expected, at least with respect to ageism. However, as the exploratory findings demonstrate, this asymmetry may be accompanied by other significant collateral effects.

### Behavioral outcomes in the workplace context

The willingness-to-hire analysis provides only tentative evidence regarding the differentiated H2 predictions and should be interpreted cautiously. At the omnibus level, Hypothesis 2 was not supported. With respect to the differentiated predictions, the pattern provided only limited descriptive support for H2a and did not support H2b. Although the overall ANOVA did not reach conventional significance, the effect-size estimate was in the medium range, suggesting that a larger sample might be needed to evaluate this outcome more conclusively. Exploratory pairwise comparisons indicated higher willingness-to-hire scores in the Einstein and Neutral Adult conditions than in the Neutral Young condition, but these comparisons should be interpreted descriptively rather than as confirmatory support, given the non-significant omnibus test. The outcome remains worth examining because applicant age is a major factor in employment discrimination globally (Kleissner and Jahn, 2021; Krings et al., 2011; Qvist and Larsen, 2025), and HR decisions are a context in which implicit ageist associations do the most harm (Kleissner and Jahn, 2020; Ratliff and Smith, 2024). However, in the present study, the willingness-to-hire findings should be regarded as provisional and in need of replication rather than as clear evidence that VR embodiment altered hiring-related judgments.

The weaker findings on hiring intentions, relative to the more robust IAT changes, are theoretically informative. They reflect the layered nature of prejudice, in which automatic associations may shift before more deliberate evaluative judgments are fully reorganized. This does not undermine the contribution; rather, it provides a realistic picture of what brief VR interventions can and cannot immediately achieve.

### Risks of negative valence avatar exposure

Exploratory analyses suggested collateral psychological effects following embodiment in the Nasrallah avatar, but these patterns should be interpreted cautiously, given that these measures were added during data collection and were not part of the original confirmatory design. One such effect was that exploratory within-condition analyses suggested a possible decrease in self-esteem scores following embodiment in the Nasrallah avatar. Because the decrease in self-esteem was assessed using an exploratory within-condition analysis and the Rosenberg Self-Esteem Scale, which is typically understood as a measure of global self-esteem, this pattern should be interpreted with caution. One possible interpretation is that embodiment in a strongly negative and morally aversive figure may have affected participants' momentary self-evaluative responses, but the present study did not include a validated state self-esteem measure and therefore cannot clearly distinguish between short-term fluctuations and broader self-evaluative change. The finding fits evidence that avatar archetype customization can influence behavior and emotional responses in virtual environments, with antihero embodiment specifically linked to increased shame when interacting with mindful virtual agents (Peña et al., 2024), and broader work on self-esteem disruption following negatively charged self-relevant experiences (Heatherton and Polivy, 1991; VanDellen et al., 2011). Low avatar comfort may also have contributed to this negative self-evaluative response.

A second exploratory outcome was an increase in implicit gender-career bias, that is, a stronger implicit association of men with career and women with family in the IAT. One possible explanation is that this effect reflected the activation of a broader aversive associative network triggered by the avatar. In the present study, the Nasrallah avatar was experienced as especially uncomfortable and was predominantly associated with aggression and negativity, suggesting that participants may have processed it primarily as a strongly negative or threat-related figure rather than simply as an older adult. If so, the activation elicited by this avatar may have extended beyond age-related meanings and may have strengthened gender-career associations as well. Alternatively, or in parallel, the distress associated with this embodiment may have increased reliance on stereotypic heuristics under threat-related cognitive conditions (Amodio, 2009). Consistent with this possibility, Lopez et al. (2019) found that male participants embodied as female avatars in a negative scenario showed increased gender-career IAT bias. Taken together, this pattern suggests that the Nasrallah avatar may have functioned more as a strongly negative cue than as an older-adult cue, although this interpretation remains tentative.

### Toward a meaning-based account of VR embodiment

The findings are consistent with the possibility that VR embodiment effects on intergroup attitudes depend not only on avatar age or appearance, but also on the meaning attached to the embodied figure. The strongest reduction in implicit ageism appeared in the Einstein condition, with a smaller reduction in the neutral adult condition, suggesting that both age-based perspective taking and avatar-specific symbolic meaning contributed to the change. One tentative reading is that two processes may be at work: a relatively general perspective-taking process associated with embodying an older adult, and a more identity-cue-based process that may become stronger when the avatar carries a highly salient positive meaning (such as wisdom). The current data, however, do not permit a direct test of these mechanisms, and alternative explanations remain possible.

The null ageism effect in the Nasrallah condition suggests that negatively valenced embodiment may not function in a straightforward way as the mirror image of positively valenced embodiment. Several interpretations remain plausible, including active disidentification from the avatar, ceiling-like baseline ageism in the sample, or competing effects of aversiveness and age-related processing. In any case, the result suggests that the effects of negative-valence embodiment may be more complex than merely reinforcing outgroup prejudice.

The exploratory findings on self-esteem and gender-career further suggest that avatar embodiment may have effects beyond the focal attitude domain. One possibility is that highly salient avatars activate broader associative or affective responses that extend beyond the target prejudice domain. If so, positively valenced avatars may have beneficial spillover effects, whereas negatively valenced avatars may carry a risk of unintended consequences. This possibility remains speculative and requires direct testing in future work.

### Additional outcomes

The neutral adult condition showed a reduction in implicit ageism in the expected direction, replicating Yee and Bailenson's (2006) findings in a workplace context. This reduction in implicit ageism demonstrates that avatar age representation alone carries perspective-taking value, with direct practical implications for organizations that need not rely on licensed famous-figure avatars.

Self-esteem did not moderate the embodiment effect, a null finding consistent with Banakou et al. (2018) and possibly explained by the domain-specificity of self-esteem's role and the likelihood that VR embodiment operates as a bottom-up, sensory-driven process independent of dispositional self-evaluation. This null finding may be contrasted with evidence from self-referential augmented-reality contexts, in which self-esteem has been shown to moderate user experience more strongly, especially when users engage with their own appearance or self-concept directly (Javornik et al., 2021; Wyszynski et al., 2025). A possible implication is that self-esteem may be more relevant in self-referential virtual contexts than in embodiment paradigms involving a different social identity. Finally, post-VR explicit ageism scores did not differ significantly across conditions at post-test, replicating the commonly observed dissociation between implicit and explicit outcomes in VR stereotype research. Consistent with dual-process theory (Strack and Deutsch, 2004), this pattern suggests that embodiment may have influenced relatively automatic associative processes without producing corresponding changes in deliberate, self-reported attitudes.

### Limitations and future directions

Several limitations should be noted. First, the sample was exclusively young, Hebrew-speaking Israeli men recruited from a single university. This homogeneity served experimental control but limits generalizability. Women experience the intersection of ageism and sexism as doubly vulnerable older workers (Ramirez et al., 2022), and gender-inclusive replication is essential. All participants were students with limited managerial experience, whereas real-world age discrimination is perpetrated by experienced HR professionals who may have more crystallized attitudes and more sophisticated impression-management skills. Field trials in which HR professionals undergo VR embodiment sessions prior to actual hiring cycles would therefore provide the strongest evidence for translational impact. A useful next step is to examine whether bias reduction generalizes across the employee lifecycle, including performance appraisal, training allocation, and promotion decisions (Harris et al., 2018).

Second, the final sample was close to the *a priori* G^*^Power target for the primary Age-IAT analysis, and smaller effective sample sizes were used for some secondary and exploratory outcomes. Therefore, the exploratory findings have limited inferential strength. Moreover, because these measures were not part of the original confirmatory design, the corresponding findings should be treated as hypothesis-generating rather than confirmatory and should be replicated in preregistered studies. Third, the study was conducted during the Israel–Hezbollah war, which almost certainly amplified the aversiveness of the Nasrallah avatar beyond what would be observed in other cultural or temporal contexts. Cross-cultural replication with locally calibrated avatar stimuli is needed (Boduroglu et al., 2006). Moreover, employing negative-valence avatars, ranging from mildly aversive to severely repulsive, to map the dose-response curve for both ageism reduction and collateral psychological outcomes is recommended. Additionally, a fully crossed avatar-age × valence factorial design was lacking, as positive and negative young avatars were not included. This limits the ability to isolate age and valence effects independently. Future work should therefore use a fully crossed avatar-age × valence design that incorporates both positive and negative young avatars alongside their older counterparts, enabling a more systematic comparison of valence effects across age groups. Fourth, the absence of longitudinal follow-up leaves the duration of effects entirely unknown, a crucial gap for the viability of interventions. Assessments administered at 1 week, 1 month, and 3 months post-embodiment are needed. Such assessments can determine the persistence of both the ageism-reducing and collateral effects.

Fifth, the study did not include a social desirability measure, which complicates the interpretation of the null findings regarding explicit ageism and willingness to hire. Future studies should incorporate a validated social desirability covariate, supplement the IAT with alternative implicit measures, and employ behavioral indicators of hiring decisions, such as resume review time and mouse-tracking, that are less susceptible to social desirability bias. Theoretically, studies should attempt to dissociate the general perspective-taking pathway from identity-cue-mediated mechanisms, and empirically test whether moral contamination and semantic activation account for the collateral effects observed following Nasrallah's embodiment.

Sixth, exploratory pre-post analyses of self-esteem were based on repeated administration of the Rosenberg Self-Esteem Scale. This scale is typically conceptualized as a measure of global rather than state self-esteem. Therefore, these pre-post analyses should be interpreted with caution.

Seventh, the coding of the Dominant Avatar Characteristic responses was conducted by a single coder. Although predefined, behaviorally anchored categories were used to reduce interpretive ambiguity, no second independent coding or inter-rater reliability statistic was available. These descriptive findings should therefore be interpreted with caution, and future studies should include independent double coding and formal agreement indices.

### Ethical implications and concluding remarks

This study raises important ethical implications for VR intervention design. The demonstration that a single VR session can measurably alter implicit attitudes without participants' awareness raises a dual-use concern: the same technology that can reduce stereotypes could be deployed to amplify them. Researchers, VR developers, and policymakers should collaborate to establish ethical standards for commercial VR content. The exploratory pattern observed in Nasrallah's condition suggests that negatively valenced avatars may pose collateral risks warranting careful ethical consideration and replication. These risks may include possible self-esteem effects and heightened gender bias, suggesting that future protocols involving negative-valence avatars include comprehensive post-session debriefing, psychological wellbeing screening, and potentially a “cleansing” procedure (e.g., brief positive-avatar embodiment). While participants were informed that the study involved a VR experience and were free to withdraw at any time, they were not specifically warned that they might be embodied in a figure as politically and morally provocative as Nasrallah. Although this was necessary for the experimental design, it raises legitimate questions about the boundaries of informed consent. Informed consent frameworks should disclose the range of avatar identities participants may encounter without revealing specific figures. Moreover, the use of avatars based on real public figures without consent raises intellectual property concerns that standard frameworks do not address, particularly for first-person embodiment. As Slater (2021) argues, the field has an ethical obligation to guard against the misuse of immersive technologies. These considerations are not obstacles to VR-based organizational interventions but essential design constraints that make such interventions ethically defensible. An interesting complementary direction is noted by Zou et al. (2024), who found that intergenerational collaboration in VR, rather than individual embodiment, produced significant reductions in both explicit and implicit age bias. Combining this framework with valenced avatar embodiment represents a promising avenue for future research.

In sum, avatar valence appears to matter for VR embodiment effects on implicit workplace ageism, though the pattern was not uniform across outcomes and should be interpreted cautiously. Positive embodiment, particularly in the Einstein condition, was associated with reduced implicit ageism, while the Nasrallah condition highlighted the complexity and potential risks of negatively valenced embodiment. The study underscores the importance of symbolic meaning in shaping embodiment effects and suggests that VR may hold promise as a tool for reducing ageism in workplace contexts, provided its effects and ethical implications are carefully evaluated.

## Data Availability

The data analyzed in this study is subject to the following licenses/restrictions: De-identified data supporting the conclusions of this article will be made available by the corresponding author upon reasonable request, subject to ethical approval and applicable institutional restrictions. Requests to access these datasets should be directed to Ehud Bodner, ehud.bodner@biu.ac.il.
