# Supplementary material for: Can avatar affective valence determine whether virtual reality embodiment reduces implicit workplace ageism?
Source: Front Psychol. 2026 Jun 30;17:1868756. doi: 10.3389/fpsyg.2026.1868756 (PMC13364684; doi:10.3389/fpsyg.2026.1868756)
Supplement: Supplementary file 1 [file Table_1.docx]

**Table S1. Rate of Dominant Embodied Avatar Characteristics by Condition**

| **Category** | **Einstein n/N (%)** | **Nasrallah n/N (%)** | **Neutral Adult n/N (%)** | **Neutral Young n/N (%)** |
| --- | --- | --- | --- | --- |
| Wisdom | 14/28 (50.0%) | 0/25 (0.0%) | 1/26 (3.8%) | 0/22 (0.0%) |
| Aggression | 0/28 (0.0%) | 6/25 (24.0%) | 0/26 (0.0%) | 0/22 (0.0%) |
| Total Positive | 18/28 (64.3%) | 3/25 (12.0%) | 7/26 (26.9%) | 4/22 (18.2%) |
| Total Negative | 2/28 (7.1%) | 10/25 (40.0%) | 5/26 (19.2%) | 1/22 (4.5%) |
| Old Age | 2/28 (7.1%) | 4/25 (16.0%) | 11/26 (42.3%) | 0/22 (0.0%) |
| Young Age | 0/28 (0.0%) | 0/25 (0.0%) | 0/26 (0.0%) | 4/22 (18.2%) |

Note. Values are n/N (%). The table summarizes the distribution of coded dominant-avatar characteristics across conditions. As described in the main text, Einstein was most strongly associated with wisdom and positive descriptors, Nasrallah with aggression and negative descriptors, and neutral conditions were primarily differentiated by age-related descriptors. Four participants in the neutral young group had missing values.
