# Supplementary material for: Can avatar affective valence determine whether virtual reality embodiment reduces implicit workplace ageism?
Source: Front Psychol. 2026 Jun 30;17:1868756. doi: 10.3389/fpsyg.2026.1868756 (PMC13364684; doi:10.3389/fpsyg.2026.1868756)
Supplement: Supplementary file 2 [file Data_Sheet_1.pdf]

Appendix Questionnaires

Presence and Embodiment scale (Adapted)

Banakou, D., Beacco, A., Neyret, S., Blasco-Oliver, M., Seinfeld, S., & Slater, M. (2020). Virtual body ownership and its consequences for implicit racial bias are dependent on social context. Royal Society Open Science, 7(12), 201848. https://doi.org/10.1098/rsos.201848

1. When I looked down at myself, I felt that the virtual body I saw was my body.
2. When I looked at myself in the mirror, I felt that the virtual body I saw was my body.
3. I felt as if the virtual body was not me.
4. I felt that the movements of the virtual body were caused by my own movements.
5. I had the feeling that I was sitting in a conference lobby.
6. I had the feeling that the experience taking place in the virtual world was really happening.

Explicit ageism - SIC scale for the workplace (Adapted)

North, M. S., & Fiske, S. T. (2013). A prescriptive intergenerational-tension ageism scale: Succession, identity, and consumption (SIC). Psychological Assessment, 25(3), 706–713. https://doi.org/10.1037/a0032367

1. Younger employees spend too much time explaining things to older employees (over age 60).
2. Older employees (over age 60) are a burden on the organizations in which they work.
3. Older employees (over age 60) are often too much of a burden on their coworkers.
4. At a certain point, the greatest benefit of older employees (over age 60) in the workplace is their early retirement.
5. Older employees (over age 60) should not accumulate so many pension benefits at the expense of younger employees' pensions.

6. Older employees (over age 60) should not receive the best positions (jobs) in the workplace.
7. The Ministry for Senior Citizens (the ministry responsible for developing programs and services for older adults) wastes taxpayers' money.
8. If older employees (over age 60) were not so resistant to learning new technologies, the organizations in which they work would probably be more prosperous.
9. Older managers (over age 60) have accumulated too much political power in the workplace compared with younger managers.
10. Most older managers (over age 60) do not know when to vacate their positions for younger managers.
11. Most older employees (over age 60) do not know when it is time to make room for the younger generation.
12. Older employees (over age 60) are often too stubborn to admit that they are no longer functioning as well as they used to.
13. Younger employees usually complete more tasks than older employees (over age 60).
14. Promotion at work should not be based on the experience of older employees (over age 60), but rather on how many tasks they are able to complete.
15. It is unfair that older employees (over age 60) make decisions on issues that will affect younger employees.
16. Older employees (over age 60) should not be employed in jobs for young people (for example, in high-tech).
17. It is inappropriate for older employees (over age 60) to be employed in jobs for young people.
18. In general, it is inappropriate for older employees (over age 60) to socialize with younger employees.
19. In general, older employees (over age 60) should not promote themselves so much on LinkedIn.
20. Older employees (over age 60) should not try to act cool.

Willingness to Hire an Adult Employee (Developed for the study)

In the following task, you are asked to imagine that you are a Human Resources manager responsible for recruiting new employees for the company in which you work. The position for

which you are required to recruit a suitable candidate is National Supervisor of Customer Service Teams in the call center of an Israeli communications company.

Below are the résumé details of one of the candidates. Please read them carefully.

Personal details:
Name: Yoav Dor
Age: 60
Address: 4 Histadrut Street, Tel Aviv
Marital status: Married + 2

Education:
1997–2000 – Bachelor's degree in Economics, The Hebrew University

Work experience:
2015–present – Central District Supervisor, Customer Service, Cellcom
2008–2015 – Tel Aviv Area Team Leader, Customer Service, Cellcom
2000–2008 – Customer Service Shift Supervisor, Bezeq
1995–2000 – Customer Service Representative, Bezeq

Military service:
Israeli Air Force

Languages:
Hebrew – Native language
English – Very good

After you finish reading, please indicate the extent to which you agree or disagree with the following statements:

1. The candidate is suitable for the position.

2. This candidate should get the position.

3. I would hire this candidate for the position.

4. I would like to work on a team with this candidate.
